# Brivaracetam and topiramate serum levels during pregnancy and delivery: a case report and a review of literature

**DOI:** 10.1186/s42466-024-00312-9

**Published:** 2024-03-21

**Authors:** Wiebke Hahn, Leona Möller, Katja Menzler, Tobias Poeplau, Uwe Wagner, Susanne Knake

**Affiliations:** 1 Department of Neurology, Philipps-University Marburg, Baldingerstraße, 35043 Marburg, Germany; 2 Department of Gynaecology, Philipps-University Marburg, Baldingerstraße, 35043 Marburg, Germany

**Keywords:** Epilepsy, Pregnancy, Drug monitoring, Brivaracetam

## Abstract

**Background:**

An increasing use of newer antiseizure medication (ASM) such as SV2A ligand brivaracetam is observed. However, data on newer antiseizure medication and therapeutic drug monitoring during pregnancy is scarce.

**Methods:**

Therapeutic drug monitoring of brivaracetam (BRV) and topiramate (TPM) serum levels were performed during pregnancy, delivery and in the umbilical cord blood at delivery in a 34-year-old female patient with severe drug-resistant epilepsy.

**Results:**

During pregnancy, the serum levels of brivaracetam and topiramate remained stable. At 39th week of pregnancy, the patient gave birth to a healthy daughter. 1.5 h after the last ASM intake, the penetration rate measured in umbilical cord blood was 45% lower for BRV and 35% lower for TPM.

**Conclusions:**

While the pharmacokinetics of topiramate are well known and its use during pregnancy should only be undertaken under special circumstances, there have been few studies on newer ASM in pregnancy such as brivaracetam. Based on our results and other case reports of BRV use during pregnancy, further studies are necessary to confirm its pharmacokinetics and safety during pregnancy.

## Background

The selective synaptic vesicle protein 2A (SV2A) ligand brivaracetam has been approved as a seizure suppressant drug in Europe since 2016. Compared to its predecessor drug levetiracetam (LEV), BRV binds to the SV2A protein with a 15- to 30-fold greater affinity without simultaneous modulation of the AMPA receptor [23]. With a significant reduction in seizures with additional treatment with BRV, various studies have demonstrated good overall tolerability of the new antiseizure medication (ASM) as well as a reduced occurrence of psychiatric undesirable drug effects such as depression and irritability compared to LEV [1, 2, 20, 23].

The risks of neurodevelopmental disorders and congenital malformations during pregnancy with antiseizure medication such as levetiracetam or lamotrigine are considered to be relatively low [13]. Due to significant fluctuations in serum levels, dosage adjustments are often necessary during pregnancy with lamotrigine and levetiracetam [5]. Although an increase in prescriptions of newer ASM such as BRV can be observed [7], little is known about the teratogenic effects of BRV and potential serum level fluctuations during pregnancy.

In a retrospective multicenter study, Steinig et al. [20] examined post-marketing effects like efficacy, retention and tolerability in 262 patients with epilepsy who received brivaracetam as add-on therapy. During the observation period, two pregnancies occurred while taking BRV. As soon as the pregnancy became known, antiseizure medication was switched back to levetiracetam in accordance with the guidelines. Both pregnancies continued after temporary use of BRV. In 2020, a first case series of three women with epilepsy treated with brivaracetam during pregnancy was reported by Paolini and colleagues: The daily dose of BRV intake ranged from 50 mg/d to 200 mg/d. While two patients received BRV as monotherapy, the third received lamotrigine (LTG) as a comedication. Minor congenital malformations such as infantile hemangioma, congenital dermal melanocytosis and ankyloglossia were observed in two of three children (see Table 1). Navarro et al. [14] reported the birth of a healthy child on BRV therapy during pregnancy in a patient with therapy-refractory juvenile myoclonic epilepsy. Landmark and colleagues [11] reported three healthy births during pregnancy with brivaracetam in two patients with epilepsy: Whereas one patient received BRV in monotherapy, the other also received perampanel and lacosamide as comedication. During pregnancy, serum levels of BRV remained stable in both patients. While the serum levels of BRV in the umbilical cord of both twins of one patient were reported as comparable to the blood concentration of their mother as measured one hour after last dose intake (infant/mother ratios: 1.1), BRV serum level in the umbilical cord of another newborn was reported as low (infant/mother ratio: 0.18) compared to its mother´s level as measured 9.5 h hours after last dose intake (see Table 1).

**Table 1:** Overview of previously reported cases of brivaracetam during pregnancy and childbirth in women with epilepsy

Authors	Cases	BRV/d	Further ASM/d	Conc. of BRV during pregnancy	Conc. of BRV in umbilical cord	Conc. of BRV postpartum)	Serum ratio (infant/mother)	Newborn malformation
Paolini et al. [15]	3	P1*: 100 mg P2: 150 mg P3: 200 mg	None None P3: LTG 450 mg	– – –	– – –	– – –	– – –	Congenital dermal melanocytosis and ankyloglossia Infantile hemangioma on thumb and back None
Landmark et al. [11]	2	P 1: 150 mg P2: 200 mg	P1: none P2: PER 8 mg, LCM 400 mg	P1: 3,8 umol/l **(0.81 mg/l) P2: 6,6 umol/l **(1.40 mg/l)	Twin 1: 4,1 umol/l **(0.87 mg/l) Twin 2: 4,2 umol/l **(0.89 mg/l) P2: 1, 2umol/l **(0.25 mg/l)	P1: 4,4 umol/l **(0.93 mg/l) P2: 6,1 umol/l **(1.29 mg/l)	1.08 1.11 0.18	None None None
Navarro [14]	1	200 mg	none	–	–	–	–	None

*P1 = Patient 1, P2 = Patient 2, BRV = brivaracetam, ASM = antiseizure medication, LTG = lamotrigine, PER = perampanel, LCM = lacosamide

**For better comparability of the data, the amount of substance was converted into weight units

## Case report

Here we report a 31-year-old right-handed female patient who presented with pregnancy while being treated with BRV and TPM. She had focal, non-aware seizures occurring several times per week. During the seizures, she felt tachycardia and dizziness and did not respond to speech.

In her past medical history, the patient reported febrile seizures that had occurred at the age of 6 years and a traumatic brain injury at the age of 11 years. The patient was medically refractory to lamotrigine (LTG) and levetiracetam (LEV). Due to the diagnosis of drug-resistant epilepsy, presurgical video-EEG monitoring was performed in 2014 and right occipital lobe epilepsy was diagnosed. A 3-Tesla cMRI showed no evidence of epileptogenic lesions. The patient refused any operative procedure. Subsequently, various antiseizure medications were given due to inadequate seizure control or side effects, such as zonisamide (ZNS), topiramate (TPM), valproate (VPA), carbamazepine (CBZ), oxcarbazepine (OXC), perampanel (PER), gabapentin (GBP) and BRV.

In December 2021, the patient reported that she was pregnant, and was already 17 weeks pregnant. She presented with BRV (200 mg/d) and TPM (200 mg/d) and a seizure frequency of one per day. Serum levels of antiseizure medications, taken approximately 6 h after the morning doses, were 2.91 mg/l for BRV and 4.7 mg/l for TPM. The patient was already taking folic acid supplementation daily during pregnancy (5 mg/d). There was no switch back to levetiracetam, as is usual during pregnancy, as the patient refused this due to ineffectiveness and intolerance to this medication in the past. Due to the advanced pregnancy, it was assumed that organogenesis had already been completed. After detailed explanation, we decided together to continue the previous antiseizure medication. We agreed on monthly outpatient follow-ups. During those, the serum levels of antiseizure medication were stable (see Table 2). Since the patient was only able to attend appointments in the early afternoon, drug levels were taken 6 h after the last dose. Seizure frequency during pregnancy was unchanged. At week 39, the patient gave spontaneous birth to a healthy daughter (Apgar score: 9/10/10). 1.5 h after the last intake of antiseizure medication, the serum levels in the umbilical cord blood were 1.97 mg/l for brivaracetam and 3.0 mg/l for topiramate. The serum ratio of BRV (infant/mother) was 0.58 and serum ratio of TPM was 0.56. One month after delivery, the patient presented to our outpatient clinic. Her serum level of BRV was relatively stable (3.32 mg/l) comparable to the serum levels during pregnancy, whereas the serum level of TPM increased (7.3 mg/l). Seizure frequency was unchanged. Eight months after delivery, the patient returned to our outpatient clinic. She had stopped breastfeeding 6 months after delivery. The daughter was healthy and appropriately developed. BRV and TPM were still taken with unchanged daily dosage. Due to recent data on the increased risk of carcinoma with daily folic acid supplementation of more than 1 mg [22], folic acid therapy was reduced to 0.8 mg daily.

**Table 2:** Blood serum concentrations of brivaracetam (BRV) and topiramate (TPM) before, during and after pregnancy

	BRV concentration (200 mg/d) Reference range: 0.5–0.9 mg/l* (mg/l)	TPM concentration (200 mg/d) Reference range: 2.0–10.0 mg/l (mg/l)
Before pregnancy	3.31	4.7
Week of pregnancy: 17	2.91	4.7
Week of pregnancy: 21	4.53	6.5
Week of pregnancy: 25	4.21	5.7
Week of pregnancy: 30	2.48	4.1
Week of pregnancy: 33 +	2.57	4.5
Week of pregnancy: 37	3.6	4.6
After pregnancy (1 month)	3.32	7.3
After pregnancy (8 month)	3.87	6.2
Umbilical cord blood	1.97	3.0

The blood was taken regularly at noon about 6 h after taking the ASM

*This reference range is validated for daily intake of 100 mg brivaracetam

## Discussion

We report a 34-year-old patient with difficult-to-treat pharmacoresistant epilepsy who gave birth to a healthy child after pregnancy with topiramate and brivaracetam. Topiramate may be associated with an increased incidence of major congenital malformations: Based on data from 7355 pregnancies in the EURAP study group, Tomson and colleagues showed that 6 of 152 pregnancies (3.9%) with topiramate lead to major congenital malformation, such as cardiac malformation or hypospadias [6, 21]. A review of the Cochrane database also showed that prenatal exposure to topiramate may lead to facial malformations [3]. A systematic review by Schmidt and colleagues [18] examining umbilical cord transmission of ASM found that antiseizure medication associated with an increased risk of congenital malformations, such as TPM, CBZ or VPA showed relevant differences among each other with respect to penetration into the fetal circulation. Regarding topiramate, the authors reported an intermediate penetration rate of topiramate into the umbilical cord with a comparatively high penetration of TPM into breast milk. Due to the risk of congenital malformations, fetal growth impairments and possible neuropsychiatric developmental disorders, the drug commission of the German medical association published a red hand letter in November 2023 with restrictions to prevent exposure to topiramate during pregnancy [4]. Although an increasing use of newer ASM like SV2A ligand brivaracetam can be observed [7, 8], evidence on newer antiseizure medication during pregnancy is scarce, particularly regarding therapeutic drug monitoring. The SV2A ligand brivaracetam is rapidly absorbed. According to previous evidence in healthy volunteers on BRV monotherapy, a dose-dependent peak in serum concentration is reached 2 h after ingestion with a plasma half-life of 9 h [16, 17, 19]. To our knowledge the study by Landmark and colleagues [11] is the first to provide pharmacokinetic data on brivaracetam in pregnancy and lactation. Landmark and colleagues determined serum concentrations 3 h after ingestion of 75 mg BRV (P1) and 9.5 h after ingestion of 100 mg BRV (P2). At P1 this was 0.81 mg/l. At P2 this was 1.4 mg/l. However, P2 was also on comedication with LCM and PER. The serum concentrations of BRV remained relatively stable in our patient, when 100 mg of BRV was taken twice daily. Since our patient was only able to attend appointments in the early afternoon, drug levels were taken at latest 6 h after the last dose intake. Compared to P1 in Landmark and colleagues, the concentration of BRV in our patient was higher despite a later blood sample. Compared to P2 in Landmark and colleagues, the concentration of BRV in the blood was also higher, with the same BRV dose but earlier blood sampling. Possible reasons for these results could be pharmacogenetic factors as well as metabolic influences of comedication with topiramate. Although there is little evidence regarding pregnancy and birth with newer ASM such as brivaracetam, the present case demonstrated that an uncomplicated pregnancy and healthy childbirth is possible.

In a questionnaire-based survey, Mann and colleagues examined the knowledge of women with epilepsy (WWE) about their disease in a multicenter study between October and December 2020 and compared these results with data from 2005. Regarding disease-specific knowledge, there were no significant group differences in the cohort between 2005 and 2020. The authors found that special life events, like pregnancy in women with epilepsy, are associated with worries and false beliefs, which may lead to a reduction in ASM during pregnancy or a delayed medical consultation. The lack of knowledge correlated with lower educational level and with an elderly age. Due to the fact, that women with epilepsy are more likely to have unplanned pregnancies [9], regular neurological-epileptological consultations should take place. In these, the change to ASM should be discussed regularly and in line with the patient`s prior knowledge if she wishes to have children. Furthermore, patients and their partners should be repeatedly informed, that a neurological consultation regarding a change or adjustment of the antiseizure medication should take place immediately after the pregnancy becomes known.

Due to the fact, that half of epilepsies begin in childhood, the transition consultation should play a key role in providing information about epilepsy and pregnancy [12]. In addition, interdisciplinary collaboration with gynecological colleagues should also be expanded. Finally, especially in the case of pregnancies under new antiseizure medications, reporting to pregnancy registries (e.g. EURAP) is necessary as well as an easier access to research databases [10].

## Conclusions

Before or at latest when patients with ASM get pregnant, counselling should be performed by neurologist or epilepsy specialists to assess pharmacotherapy. Because there is little evidence regarding pregnancy with newer antiseizure medication, particularly regarding ASM concentrations, data donation to the pregnancy registries is important. Further data on the safety of brivaracetam during pregnancy are needed.

## Data Availability

The data that support the findings of this study are available from the corresponding author upon reasonable request.

## Abbreviations

AMPA: α-Amino-3-hydroxy-5- methyl-4-isoxazolepropionic acid
ASM: Antiseizure medication
BRV: Brivaracetam
CBZ: Carbamazepine
cMRI: Cranial magnetic resonance imaging
EEG: Electroencephalography
GBP: Gabapentin
LCM: Lacosamide
LEV: Levetiracetam
LTG: Lamotrigine
OXC: Oxcarbazepine
PER: Perampanel
SV2A: Synaptic vesicle protein 2A
TPM: Topiramate
VPA: Valproate
ZNS: Zonisamide
